# Frailty as a determinant of surgical morbidity, adjuvant therapy feasibility, and survival in octogenarian patients undergoing gastrointestinal cancer resection

**DOI:** 10.1186/s12877-026-07173-8

**Published:** 2026-02-17

**Authors:** Ayşegül Dumludağ, Deniz Öcal, Mehmet Torun

**Affiliations:** 1 Department of Medical Oncology, Erzurum City Hospital, Erzurum, Turkey; 2 Department of Gastrointestinal Surgery, Erzurum City Hospital, Erzurum, Turkey

**Keywords:** Frailty, Elderly, Gastric cancer, Colorectal cancer, Octogenarian, Modified frailty index

## Abstract

**Background:**

Frailty has emerged as a critical determinant of postoperative morbidity, mortality, and survival in elderly cancer patients. However, evidence focusing specifically on octogenarian patients undergoing major gastrointestinal cancer surgery remains limited.

**Methods:**

This retrospective cohort study included 274 patients aged ≥ 80 years who underwent curative-intent colorectal or gastric cancer surgery at Erzurum City Hospital between 2015 and 2025. Patients were stratified into Low (0–1), Medium (2), and High (≥ 3) frailty groups using the modified Frailty Index-5 (mFI-5). Perioperative outcomes, adjuvant therapy feasibility, overall survival (OS), and recurrence-free survival (RFS) were evaluated and compared across frailty categories.

**Results:**

Of the 274 patients, 153 (55.8%) were classified as low frailty, 81 (29.6%) as medium frailty, and 40 (14.6%) as high frailty. Increasing frailty severity was associated with higher rates of major postoperative complications (12.5%, 27.8%, and 46.9%) and higher observed 90-day mortality (13.7%, 14.8%, and 37.5%). Median length of hospital stay differed across frailty groups and was 9.0 (IQR 6.0–12.0) days in Low frailty, 8.0 (IQR 6.0–12.0) days in Medium frailty, and 12.0 (IQR 8.0–15.0) days in High frailty patients. In multivariable analyses, high frailty was independently associated with higher 90-day major morbidity (adjusted OR 8.92, 95% CI 2.09–38.12; *p* = 0.003) and poorer OS (adjusted HR 3.70, 95% CI 1.88–7.28; *p* < 0.001). High frailty was also associated with poorer RFS in adjusted analyses (adjusted HR 3.85, 95% CI 1.41–10.50; *p* = 0.008).

**Conclusion:**

Preoperative frailty, as assessed by the mFI-5, is associated with increased perioperative morbidity and worse survival outcomes in octogenarian patients undergoing colorectal or gastric cancer surgery. Routine frailty assessment may support risk stratification and shared decision-making in this vulnerable population.

## Introduction

The global population is aging at an unprecedented rate, and the number of individuals aged 80 years and older is expected to increase markedly in the coming decades [1]. Colorectal and gastric cancers are among the most prevalent malignancies in this population, posing significant challenges for clinicians [2]. While surgery remains the cornerstone of curative treatment, octogenarian patients carry higher risks of perioperative morbidity and mortality compared with younger cohorts [3]. Chronological age alone, however, is not a sufficient predictor of surgical risk because it fails to account for comorbidities, nutritional status, and physiological reserve [4]. Frailty, defined as a multidimensional syndrome of decreased physiological capacity and resilience, has emerged as a more accurate predictor of outcomes than age itself [5].

Frailty results from cumulative deficits across multiple systems, leading to increased vulnerability to stressors and a diminished ability to recover from surgical trauma [6]. Several frailty assessment instruments have been developed, yet many are time-consuming and impractical for routine use [7]. The Modified Frailty Index-5 (mFI-5) is a simple, validated, and widely adopted measure that includes five comorbidity-based components, providing a practical approach to risk stratification [8]. Recent studies have confirmed that frailty measured by mFI-5 is associated with severe morbidity, prolonged length of stay, and mortality in patients undergoing major gastrointestinal surgery [9–11]. In gastric cancer, frailty has been linked to increased postoperative complications, delayed recovery, and impaired overall survival [12]. Similarly, in colorectal cancer, frail patients have demonstrated higher risks of both short-term mortality and long-term recurrence [13, 14]. Both colorectal and gastric cancers represent common gastrointestinal malignancies in octogenarians and frequently require major abdominal resections; therefore, evaluating frailty as a cross-cutting vulnerability marker across these tumor sites may offer clinically pragmatic risk stratification in real-world surgical oncology practice.

Frailty not only influences perioperative outcomes but also affects the feasibility of adjuvant therapy. Several studies have suggested that frail patients are less likely to initiate or complete chemotherapy, which may compromise oncological outcomes [15]. Elderly patients are often excluded from randomized controlled trials, resulting in a paucity of high-quality data to guide clinical decision-making in this age group [16]. Moreover, evidence regarding octogenarian patients remains scarce, as most existing studies have focused on septuagenarians [17] and have rarely examined adjuvant treatment feasibility together with long-term oncological outcomes in this specific age group.

Octogenarians frequently present with higher comorbidity burdens, sarcopenia, and malnutrition, all of which are closely linked to frailty [18]. Therefore, understanding the prognostic impact of frailty in this specific age group is essential. Given the evolving perioperative and oncological strategies over the last decade, it is also important to clarify whether frailty remains prognostic across contemporary real-world practice.

In Türkiye and other middle-income countries, the rapid growth of the elderly population presents additional challenges for perioperative management and resource allocation [19]. Incorporating frailty assessment into preoperative evaluation may aid in patient selection, guide multidisciplinary discussions, and optimize treatment strategies [20]. Furthermore, frailty assessment could facilitate early interventions such as nutritional support and physiotherapy to improve surgical resilience [21]. Despite growing evidence, the relationship between frailty, perioperative outcomes, adjuvant therapy feasibility, and survival in octogenarians with colorectal or gastric cancer remains underexplored.

The present study aimed to evaluate the association of mFI-5–defined frailty with perioperative morbidity, 90-day mortality, adjuvant therapy initiation and completion, and long-term survival including both short- and long-term outcomes among patients aged 80 years and older undergoing curative-intent colorectal or gastric cancer surgery. We hypothesized that increasing frailty severity would be associated with higher perioperative complications and mortality, lower feasibility of adjuvant treatment, and significantly shorter overall and recurrence-free survival.

## Materials and methods

### Study design and patient selection

This investigation was designed as a single-center, retrospective cohort study conducted in the Department of Gastrointestinal Surgery and Medical Oncology, Erzurum City Hospital (Türkiye). All consecutive patients aged 80 years or older who underwent curative-intent resection for histologically confirmed colorectal or gastric adenocarcinoma between January 1, 2015 and September 15, 2025 were screened using multiple institutional databases, including the electronic medical record (EMR), operative logs, anesthesia charts, oncology day-care records, and the pathology information system. Following predefined eligibility criteria, a total of 274 patients were included in the final study cohort.

The study protocol was reviewed and approved by the Erzurum Faculty of Medicine Scientific Research Ethics Committee (approval date: 12 November 2025; decision number: 284). All procedures were conducted in accordance with the Declaration of Helsinki. The requirement for informed consent was waived due to the retrospective nature of the study and the use of anonymized data, in line with institutional regulations.

### Eligibility criteria

Inclusion criteria were:

- Age ≥ 80 years at the time of surgery;
- Histologically confirmed colorectal or gastric adenocarcinoma;
- Curative-intent resection (R0/R1 planned) performed as the index operation at our institution;
- Availability of essential perioperative and follow-up data.

Exclusion criteria consisted of:

- Palliative procedures such as bypass surgery or diverting stoma without tumor resection;
- Missing or irretrievable data for the primary study endpoints;
- Inadequate early postoperative follow-up (< 30 days).

### Data sources and abstraction process

Two trained investigators independently abstracted data using a pre-specified codebook and a locked extraction template designed to mirror the statistical analysis dataset. To ensure data accuracy and consistency, 10% of records were randomly re-audited by an independent reviewer, with discrepancies resolved by consensus involving a senior surgeon.

### Baseline variables

The following variables were extracted:

- Demographics: age (years), sex, and body mass index (BMI, kg/m²);
- Physiological status: ASA physical status classification (I–IV) and ECOG performance status (0–4);
- Comorbidity burden: Charlson Comorbidity Index (CCI);
- Laboratory parameters: preoperative serum albumin (g/dL) and hemoglobin (g/dL), measured within 14 days before surgery.

### Frailty assessment

Frailty was assessed using the Modified Frailty Index-5 (mFI-5), which incorporates five comorbidity-based variables: diabetes mellitus, hypertension, chronic pulmonary disease, congestive heart failure, and dependence in activities of daily living. Each present condition was assigned one point, yielding a total score ranging from 0 to 5. Patients were categorized as having Low frailty (0–1), Medium frailty (2), or High frailty (≥ 3).

Individual mFI-5 components were available at the time of index surgery but were not consistently documented in a structured format suitable for reliable retrospective extraction; therefore, analyses were performed using the composite mFI-5 score and categorized frailty groups.

### Tumor and treatment characteristics

Oncological variables included tumor site (colorectal vs. gastric), anatomical subsite, AJCC pathological stage, neoadjuvant treatment status, urgency of surgery (elective vs. emergency), surgical approach (open vs. minimally invasive), type of resection, and margin status (R0/R1).

### Perioperative outcomes

Short-term perioperative outcomes included:

- Requirement for intensive care unit (ICU) admission;
- Length of hospital stay (LOS, days);
- Surgical-site infection (SSI), defined according to CDC criteria;
- Anastomotic leak, confirmed clinically or radiologically;
- Reoperation within 30 days;
- Hospital readmission within 30 days;
- All-cause 90-day mortality.

Major postoperative complications were defined as Clavien–Dindo grade III or higher. Ninety-day major morbidity was defined as the occurrence of at least one Clavien–Dindo grade ≥ III complication within 90 days after surgery.

### Adjuvant therapy and oncological outcomes

Eligibility for adjuvant therapy was determined by multidisciplinary tumor board recommendations, with stage III patients and selected high-risk stage II patients typically considered. Adjuvant treatment variables included eligibility, timely initiation (≤ 56 days after surgery), treatment completion, and reasons for discontinuation.

### Definition of long-term outcomes

Overall survival (OS) was defined as the interval from the date of surgery to death from any cause. Recurrence-free survival (RFS) was defined as the interval from surgery to radiologically or histologically confirmed recurrence or death, whichever occurred first.

### Statistical analysis

Continuous variables were assessed for normality and summarized as mean ± standard deviation (SD) or median with interquartile range (IQR), as appropriate. Categorical variables were expressed as counts and percentages. Comparisons across frailty categories were performed using ANOVA or Kruskal–Wallis tests for continuous variables and χ² or Fisher’s exact tests for categorical variables.

The primary analysis evaluated the association between frailty category and 90-day major morbidity using multivariable logistic regression adjusted for clinically relevant confounders, including age, sex, BMI, ASA, ECOG, CCI, tumor site, AJCC stage, urgency of surgery, surgical approach, serum albumin, hemoglobin, neoadjuvant therapy, and resection type. Adjusted odds ratios (aOR) with 95% confidence intervals (CI) were reported.

Survival outcomes were analyzed using Kaplan–Meier methods with log-rank tests. Multivariable Cox proportional hazards models were used to estimate adjusted hazard ratios (aHR) for OS and RFS. Proportional hazards assumptions were verified using Schoenfeld residuals. These multivariable Cox models were adjusted for age, sex, BMI, ASA physical status, ECOG performance status, Charlson Comorbidity Index, tumor site, AJCC stage, urgency of surgery, surgical approach, serum albumin, hemoglobin level, neoadjuvant therapy, and frailty category.

To evaluate adjuvant therapy feasibility, multivariable logistic regression analyses and landmark analyses at 56 days post-surgery were performed to minimize immortal-time bias.

All statistical analyses were conducted using Python software (version 3.11). Multivariable regression analyses were performed using the statsmodels library (version 0.14.0), and survival analyses were conducted using the lifelines package (version 0.27.8). A two-sided pvalue < 0.05 was considered statistically significant.

## Results

### Patient population and baseline characteristics

**Fig. 1:**
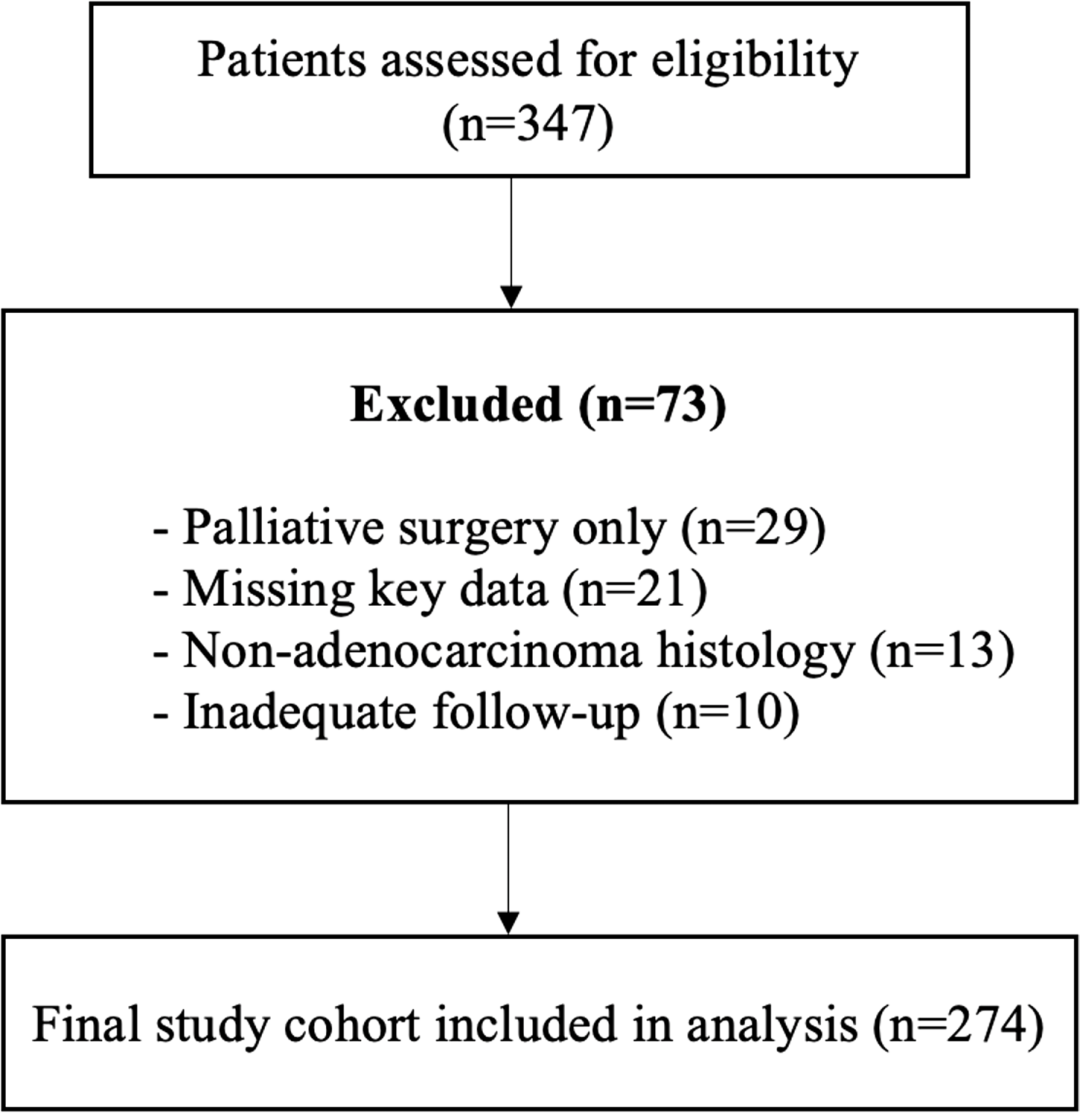
Flow diagram of patient selection

A total of 274 octogenarian patients were included in the final analysis. Of these patients, 154 (56.2%) had colorectal cancer and 120 (43.8%) had gastric cancer. Frailty distribution was comparable between tumor types. According to frailty status, 153 patients (55.8%) were classified as Low frailty, 81 patients (29.6%) as Medium frailty, and 40 patients (14.6%) as High frailty (Table 1). A detailed flow diagram illustrating patient screening, exclusion reasons, and final cohort selection is provided in Fig. 1. The overall median age was 83 years (interquartile range [IQR], 81–84) and was comparable across frailty groups.

**Table 1:** Baseline characteristics by frailty category

Variable	Low frailty (*n* = 153)	Medium frailty (*n* = 81)	High frailty (*n* = 40)
Age, mean ± SD (years)	83.35 ± 2.84	82.52 ± 2.36	83.10 ± 2.59
Male sex, n (%)	86 (56.2)	35 (43.2)	17 (42.5)
BMI, mean ± SD (kg/m²)	25.53 ± 3.99	25.42 ± 3.86	25.78 ± 3.58
ASA ≥ 3, n (%)	105 (68.6)	81 (100.0)	40 (100.0)
ECOG ≥ 2, n (%)	49 (32.0)	36 (44.4)	40 (100.0)
Charlson Comorbidity Index, median (IQR)	1.00 (1.00–2.00)	3.00 (2.00–3.00)	4.00 (3.00–4.25)
Albumin, mean ± SD (g/dL)	3.59 ± 0.41	3.44 ± 0.39	3.24 ± 0.40
Hemoglobin, mean ± SD (g/dL)	12.20 ± 1.35	12.10 ± 1.64	11.72 ± 1.95

*Abbreviations*:* ASA* American Society of Anesthesiologists, *ECOG* Eastern Cooperative Oncology Group, *CCI* Charlson Comorbidity Index

### Perioperative outcomes

Elective surgery was performed in 133 (86.9%) Low, 69 (85.2%) Medium, and 28 (70.0%) High frailty patients. An open surgical approach was more frequently used in High frailty patients (32 [80.0%]) compared with Medium (49 [60.5%]) and Low frailty groups (77 [50.3%]). ICU admission was required in 31 (20.3%) Low, 14 (17.3%) Medium, and 21 (52.5%) High frailty patients. (Table 2). Perioperative outcomes stratified by frailty category are summarized in Table 2.

**Table 2:** Perioperative outcomes by frailty category

Variable	Low frailty (*n* = 153)	Medium frailty (*n* = 81)	High frailty (*n* = 40)
Elective surgery, n (%)	133 (86.9)	69 (85.2)	28 (70.0)
Open approach, n (%)	77 (50.3)	49 (60.5)	32 (80.0)
ICU admission, n (%)	31 (20.3)	14 (17.3)	21 (52.5)
LOS, median (IQR), days	9.00 (6.00–12.00)	8.00 (6.00–12.00)	12.00 (8.00–15.00)
Anastomotic leak, n (%)	10 (6.5)	6 (7.4)	4 (10.0)
Surgical-site infection, n (%)	11 (7.2)	8 (9.9)	5 (12.5)
Reoperation, n (%)	9 (5.9)	6 (7.4)	4 (10.0)
Readmission, n (%)	31 (20.3)	13 (16.0)	4 (10.0)
90-day mortality, n (%)	21 (13.7)	12 (14.8)	15 (37.5)

*Abbreviations*: *ICU* Intensive care unit, *LOS* Length of stay, *SSI* Surgical-site infection

**Fig. 2:**
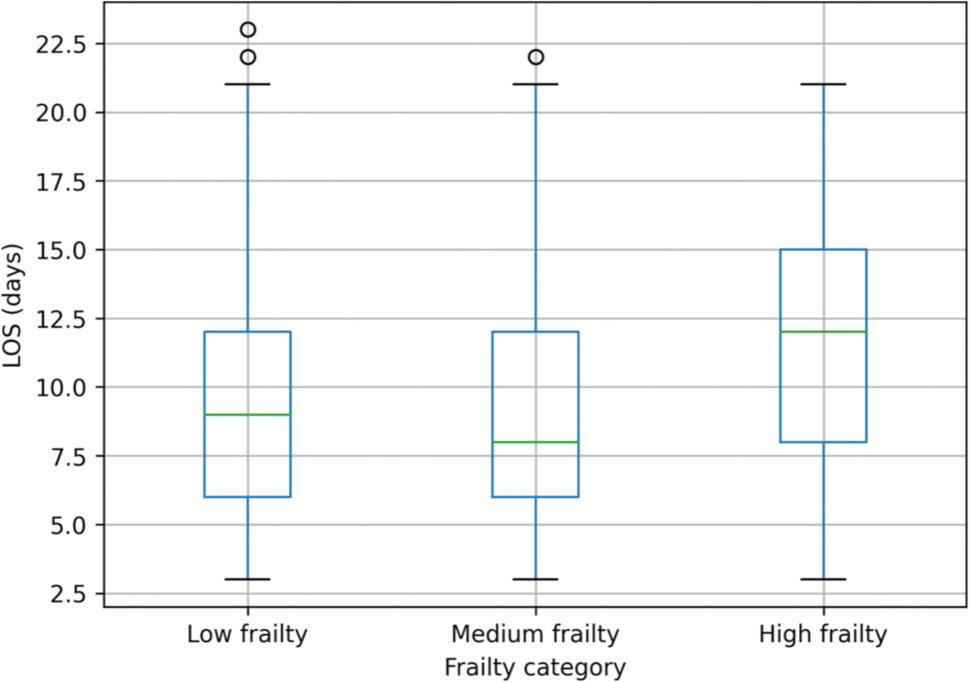
Median length of hospital stay by frailty category. Median length of hospital stay (LOS) stratified by frailty category (Low: 9.0 days; Medium: 8.0 days; High: 12.0 days)

Median length of hospital stay differed across frailty groups and was 9.0 (IQR 6.0–12.0) days in Low frailty, 8.0 (IQR 6.0–12.0) days in Medium frailty, and 12.0 (IQR 8.0–15.0) days in High frailty patients (Table 2; Fig. 2).

Postoperative anastomotic leakage occurred in 10 (6.5%) Low, 6 (7.4%) Medium, and 4 (10.0%) High frailty patients Surgical-site infection was observed in 11 (7.2%) Low, 8 (9.9%) Medium, and 5 (12.5%) High frailty patients. Reoperation rates were highest in the High frailty group (4 [10.0%]) compared with Medium (6 [7.4%]) and Low frailty patients (9 [5.9%]) (Table 2).

**Fig. 3:**
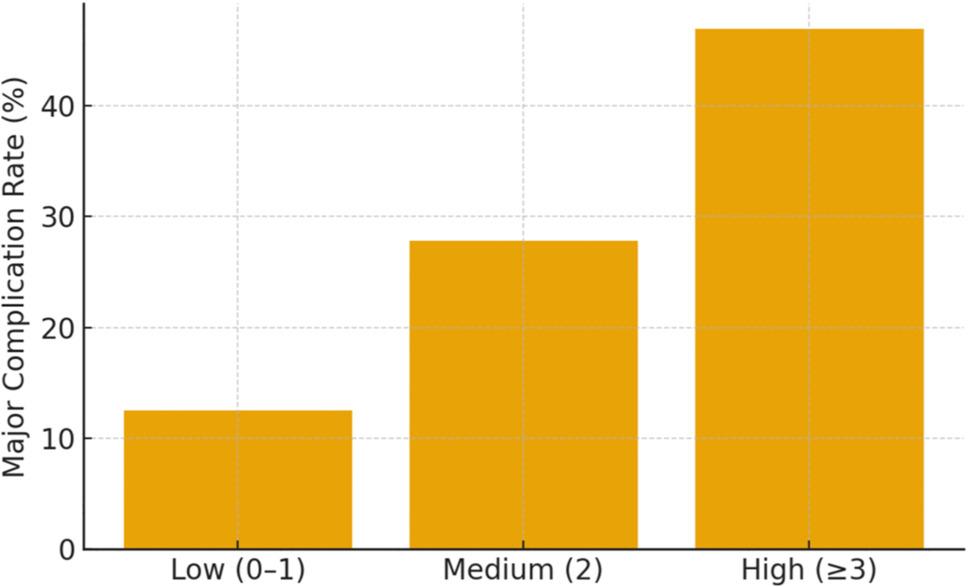
Ninety-day major complication rates by frailty category

Major complications within 90 days increased markedly with frailty severity, occurring in 19 (12.5%) Low, 22 (27.8%) Medium, and 19 (46.9%) High frailty patients (Fig. 3).

### Ninety-day mortality

**Fig. 4:**
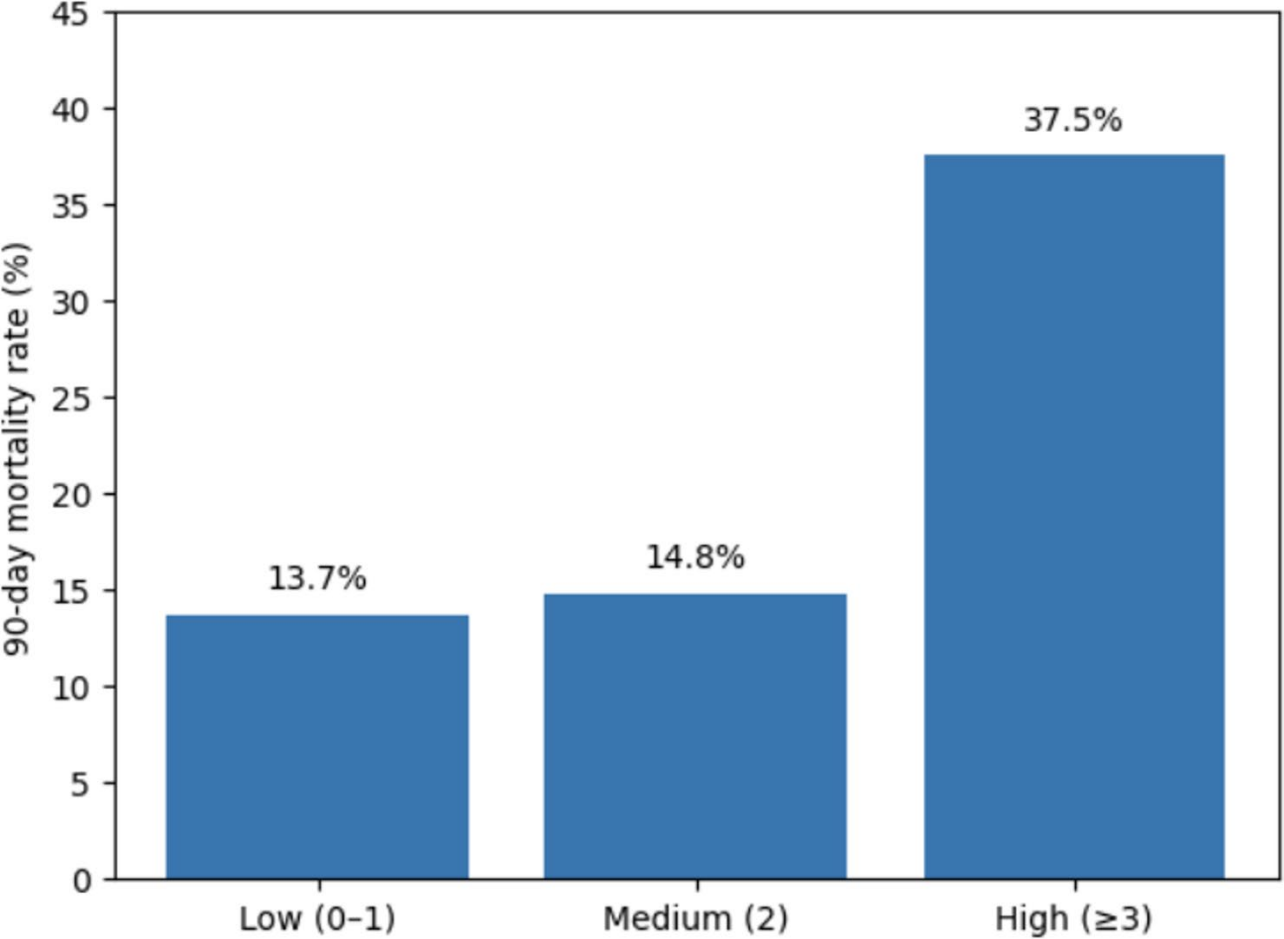
Ninety-day mortality according to frailty category

Ninety-day mortality was observed in 21 (13.7%) patients in the Low frailty group, 12 (14.8%) in the Medium frailty group, and 15 (37.5%) in the High frailty group (Table 2; Fig. 4).

### Adjuvant therapy and survival outcomes

Among patients eligible for adjuvant therapy, eligibility was confirmed in 101 (66.0%) Low, 54 (66.7%) Medium, and 32 (80.0%) High frailty patients. Timely initiation of adjuvant therapy (≤ 56 days) was achieved in 57 (56.4%) Low, 32 (59.3%) Medium, and only 4 (12.5%) High frailty patients. Completion of planned adjuvant therapy occurred in 24 (23.8%) Low, 10 (18.5%) Medium, and 2 (6.3%) High frailty patients (Table 3).

**Table 3:** Adjuvant therapy and Long-Term outcomes by frailty category

Variable	Low frailty (*n* = 153)	Medium frailty (*n* = 81)	High frailty (*n* = 40)
Eligible for adjuvant therapy, n (%)	101 (66.0)	54 (66.7)	32 (80.0)
Timely initiation (≤ 56 days), n (%)	57 (56.4)	32 (59.3)	4 (12.5)
Completion of planned therapy, n (%)	24 (23.8)	10 (18.5)	2 (6.3)
OS, median (IQR), months	22.70 (5.60–54.40)	24.10 (6.10–39.90)	2.45 (1.08–14.80)
RFS, median (IQR), months	10.80 (2.80–28.10)	12.30 (2.50–30.10)	2.30 (0.97–5.45)

*Abbreviations*: *OS* Overall survival, *RFS* Recurrence-free survival

**Fig. 5:**
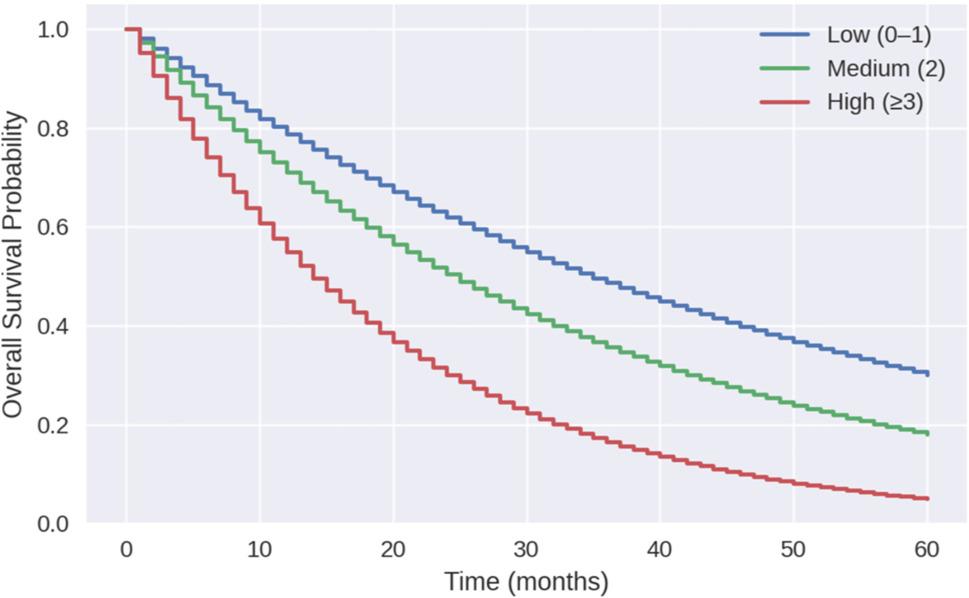
Kaplan–Meier curves for overall survival stratified by frailty category

The median overall survival (OS) for the entire cohort was 18.6 months. Stratified by frailty, median OS was 22.7 months in Low frailty patients, 24.1 months in Medium frailty patients, and 2.45 months in High frailty patients. Median recurrence-free survival (RFS) followed a similar pattern (10.8, 12.3, and 2.3 months, respectively) (Table 3). Kaplan–Meier curves demonstrated worse OS with increasing frailty severity (Fig. 5).

### Multivariable analyses

In multivariable logistic regression analysis, high frailty (mFI-5 ≥ 3) remained independently associated with increased odds of 90-day major morbidity (Clavien–Dindo ≥ III) (adjusted OR 8.92, 95% CI 2.09–38.12; *p* = 0.003) (Table 4).

**Table 4:** Multivariable logistic regression for 90-day major morbidity (Clavien–Dindo ≥ III)

Variable	aOR	95% CI	*P* value
Age (years)	0.934	0.846–1.031	0.176
Body mass index (kg/m²)	0.974	0.911–1.041	0.435
ASA physical status	0.868	0.496–1.519	0.620
ECOG performance status	1.162	0.753–1.793	0.498
Charlson Comorbidity Index	0.825	0.597–1.139	0.241
Albumin (g/dL)	1.203	0.628–2.305	0.578
Hemoglobin (g/dL)	1.033	0.869–1.227	0.712
Medium frailty (mFI-5 = 2)	1.424	0.608–3.333	0.416
High frailty (mFI-5 ≥ 3)	8.922	2.088–38.120	0.003
Male sex	1.412	0.839–2.376	0.194
Gastric cancer	1.140	0.673–1.931	0.626
AJCC stage II	0.684	0.273–1.710	0.416
AJCC stage III	1.107	0.455–2.691	0.823
AJCC stage IV	0.892	0.310–2.566	0.832
Emergency surgery	1.518	0.713–3.230	0.279
Laparoscopic approach	0.962	0.551–1.678	0.891
Robotic approach	0.575	0.104–3.172	0.525
Neoadjuvant therapy (yes)	1.000	0.545–1.836	0.999

*Abbreviations*: *aOR* adjusted odds ratio, *CI* Confidence interval, *ASA* American Society of Anesthesiologists, *ECOG* Eastern Cooperative Oncology Group, *AJCC* American Joint Committee on Cancer, *mFI-5* 5-item Modified Frailty Index

Reference categories: Low frailty (mFI-5 = 0–1); AJCC stage I; Open surgery

In adjusted Cox proportional hazards models, high frailty was independently associated with poorer overall survival (adjusted HR 3.70, 95% CI 1.88–7.28; *p* < 0.001) (Table 5) and poorer recurrence-free survival (adjusted HR 3.85, 95% CI 1.41–10.50; *p* = 0.008) (Table 6).

**Table 5:** Multivariable Cox proportional hazards model for overall survival

Variable	aHR	95% CI	*P* value
Age (years)	0.988	0.943–1.035	0.619
Body mass index (kg/m²)	1.013	0.978–1.049	0.468
ASA physical status	0.837	0.635–1.103	0.206
ECOG performance status	0.922	0.740–1.149	0.469
Charlson Comorbidity Index	0.984	0.839–1.154	0.839
Albumin (g/dL)	0.987	0.718–1.355	0.934
Hemoglobin (g/dL)	1.009	0.925–1.100	0.843
Medium frailty (mFI-5 = 2)	1.445	0.964–2.168	0.075
High frailty (mFI-5 ≥ 3)	3.701	1.882–7.278	< 0.001
Male sex	0.992	0.770–1.279	0.953
Gastric cancer (vs. colorectal)	0.923	0.709–1.201	0.550
AJCC stage II	1.338	0.838–2.138	0.223
AJCC stage III	2.256	1.429–3.562	< 0.001
AJCC stage IV	3.464	2.017–5.949	< 0.001
Emergency surgery	0.803	0.558–1.155	0.237
Laparoscopic approach	1.028	0.781–1.355	0.842
Robotic approach	1.640	0.776–3.464	0.195
Neoadjuvant therapy (yes)	0.995	0.740–1.339	0.974

*Abbreviations*: *aHR* adjusted hazard ratio, *CI* Confidence interval, *ASA* American Society of Anesthesiologists, *ECOG* Eastern Cooperative Oncology Group, *AJCC* American Joint Committee on Cancer, *mFI-5* 5-item Modified Frailty Index

Reference categories: Low frailty (mFI-5 = 0–1); AJCC stage I; colorectal cancer; open surgery

**Table 6:** Multivariable Cox proportional hazards model for recurrence-free survival

Variable	aHR	95% CI	P value
Age (years)	0.973	0.906–1.046	0.462
Body mass index (kg/m²)	0.979	0.928–1.034	0.450
ASA physical status	0.845	0.564–1.265	0.412
ECOG performance status	0.836	0.600–1.165	0.290
Charlson Comorbidity Index	0.927	0.738–1.166	0.518
Albumin (g/dL)	0.843	0.531–1.339	0.470
Hemoglobin (g/dL)	1.096	0.960–1.250	0.175
Medium frailty (mFI-5 = 2)	1.351	0.746–2.450	0.321
High frailty (mFI-5 ≥3)	3.851	1.413–10.500	0.008
Male sex	0.994	0.687–1.439	0.976
Gastric cancer (vs colorectal)	1.319	0.905–1.922	0.149
AJCC stage II	1.216	0.609–2.428	0.579
AJCC stage III	3.401	1.699–6.811	<0.001
AJCC stage IV	6.297	2.787–14.224	<0.001
Emergency surgery	0.755	0.426–1.338	0.336
Laparoscopic approach	1.433	0.972–2.112	0.069
Robotic approach	0.805	0.236–2.738	0.728
Neoadjuvant therapy (yes)	1.305	0.843–2.018	0.232

﻿*Abbreviations*:* aHR* adjusted hazard ratio, *CI* Confidence interval, *ASA* American Society of Anesthesiologists, *ECOG* Eastern Cooperative Oncology Group, *AJCC* American Joint Committee on Cancer, *mFI-5* 5-item Modified Frailty Index.

Reference categories: Low frailty (mFI-5 = 0–1); AJCC stage I; colorectal cancer; open surgery

Among patients eligible for adjuvant therapy, 90-day major postoperative complications were strongly associated with reduced odds of timely initiation of adjuvant therapy (≤ 56 days) (adjusted OR 0.18, 95% CI 0.09–0.39; *p* < 0.001). High frailty showed a trend toward delayed adjuvant initiation but did not reach statistical significance after multivariable adjustment (adjusted OR 0.14, 95% CI 0.02–1.35; *p* = 0.089) (Table 7).

**Table 7:** Multivariable logistic regression for timely adjuvant therapy initiation (≤ 56 days) among eligible patients

Variable	aOR	95% CI	*P* value
Age (years)	0.844	0.722–0.987	0.034
Body mass index (kg/m²)	0.978	0.895–1.068	0.618
ASA physical status	1.194	0.556–2.562	0.650
ECOG performance status	1.510	0.834–2.733	0.174
Charlson Comorbidity Index	0.796	0.508–1.248	0.320
Albumin (g/dL)	0.935	0.362–2.417	0.890
Hemoglobin (g/dL)	0.978	0.766–1.248	0.860
90-day major postoperative complication (Clavien–Dindo ≥ III)	0.183	0.087–0.388	< 0.001
Medium frailty (mFI-5 = 2)	1.083	0.296–3.957	0.904
High frailty (mFI-5 ≥ 3)	0.143	0.015–1.347	0.089
Male sex	0.799	0.388–1.648	0.544
Gastric cancer (vs. colorectal)	1.033	0.503–2.123	0.929
Emergency surgery	2.462	0.935–6.485	0.068
Laparoscopic approach	2.292	1.030–5.098	0.042
Robotic approach	4.249	0.298–60.514	0.286
Neoadjuvant therapy (yes)	1.378	0.583–3.254	0.465

*Abbreviations*: *aOR* adjusted odds ratio, *CI* Confidence interval, *ASA* American Society of Anesthesiologists, *ECOG* Eastern Cooperative Oncology Group, *AJCC* American Joint Committee on Cancer, *mFI-5* 5-item Modified Frailty Index

Reference categories: Low frailty (mFI-5 = 0–1); no major postoperative complication; colorectal cancer; open surgery

## Discussion

This study demonstrated that frailty, as measured by the mFI-5, is a clinically meaningful determinant of both perioperative and long-term outcomes in octogenarian patients undergoing curative-intent surgery for colorectal and gastric cancers. The results revealed a clear gradient, with patients in the high frailty group experiencing more postoperative morbidity, higher observed 90-day mortality, reduced feasibility of adjuvant therapy delivery, and markedly worse survival compared with patients in the low frailty group. These findings support the concept that frailty provides prognostic information beyond chronological age alone for risk prediction in elderly surgical oncology populations [5, 9].

Major postoperative complications increased progressively with frailty severity, with 46.9% of high frailty patients experiencing Clavien–Dindo grade III or higher morbidity. Anastomotic leaks and surgical-site infections were also more frequent in frail patients, consistent with previous studies reporting impaired wound healing, reduced functional reserve, and increased infection risk in this subgroup [11, 12]. Although absolute differences in individual complications were modest, the overall perioperative vulnerability was substantially amplified with increasing frailty severity. Consistent with these findings, reoperation rates were also numerically higher in the high frailty group, supporting increased susceptibility to clinically significant postoperative events in frail octogenarians.

Length of stay differed across frailty groups, and high frailty patients experienced a longer median hospital stay than low and medium frailty patients, consistent with earlier reports linking frailty to prolonged recovery and increased healthcare utilization [10]. These findings underscore the economic and resource burden of frailty and highlight the potential value of targeted strategies such as prehabilitation, nutritional optimization, and enhanced recovery pathways that may be tailored to vulnerable older adults [20, 21].

A stepwise increase in 90-day mortality was observed across frailty categories, with high frailty patients demonstrating substantially higher observed short-term mortality compared with low frailty patients. This mirrors existing evidence in gastric and colorectal cancer surgery, where frailty has consistently emerged as a predictor of early postoperative death [12, 14]. The marked rise in observed short-term mortality in frail patients likely reflects a combination of postoperative morbidity, competing non-cancer mortality risks, and limited physiological resilience [6]. Given that frailty is closely linked to comorbidity burden and reduced reserve, early postoperative deaths may contribute disproportionately to survival estimates in the highest frailty subgroup.

Equally important was the observed association between frailty and adjuvant therapy delivery. High frailty patients were far less likely to initiate chemotherapy within 56 days and had markedly lower completion rates compared with fitter patients. Previous research suggests that frailty-related intolerance, postoperative recovery constraints, treatment toxicity, and patient preference may all contribute to reduced chemotherapy delivery in older adults [15]. Our findings extend these observations by quantifying this limitation in a real-world cohort of octogenarians. However, in multivariable analyses among eligible patients, timely adjuvant initiation appeared to be more strongly influenced by postoperative major morbidity than frailty itself, emphasizing the central role of postoperative complications as a barrier to adjuvant treatment delivery.

Survival analyses confirmed frailty as an independent predictor of both overall and recurrence-free survival. Median overall survival in the high frailty group was 2.45 months, compared with more than 22 months in low frailty patients. While this striking difference underscores the prognostic value of frailty, the extremely poor survival in the highest frailty subgroup should be interpreted cautiously, as it may reflect residual confounding, selection factors, and a disproportionate contribution of early postoperative mortality. Accordingly, future studies may benefit from sensitivity approaches such as landmark analyses excluding early postoperative deaths or stratified analyses by surgical urgency and margin status to further contextualize this association.

Kaplan–Meier curves demonstrated early separation, suggesting that frailty influences outcomes soon after surgery and may continue to shape longer-term prognosis.

The clinical implications of these findings are substantial. Preoperative frailty assessment should be integrated into routine surgical oncology practice, particularly for octogenarians. Identifying frail patients may support individualized decision-making regarding perioperative optimization, postoperative monitoring, and the intensity and timing of adjuvant treatments. Importantly, frailty assessment should not be used to categorically deny curative-intent treatment; rather, it should facilitate shared decision-making by aligning treatment intensity with patient resilience and recovery capacity. Frailty evaluation may also complement multidisciplinary tumor board discussions by anticipating adjuvant feasibility and supporting early supportive interventions.

This study evaluated patients with colorectal and gastric cancers in a combined cohort. Although these malignancies differ in surgical complexity, recurrence patterns, and adjuvant paradigms, they represent common gastrointestinal cancers in octogenarians that frequently require major abdominal resections. Therefore, assessing frailty as a cross-cutting vulnerability marker across these tumor sites offers pragmatic applicability in real-world surgical oncology. Notably, tumor site was included as an adjustment variable in multivariable models, suggesting that the prognostic role of frailty was not solely driven by one tumor type. Nevertheless, future studies should consider tumor-specific subgroup or interaction analyses to confirm the consistency of frailty-associated risk across colorectal and gastric cancer populations.

An additional important consideration is the long inclusion period (2015–2025), during which changes in surgical techniques, perioperative pathways (including ERAS implementation), and oncological strategies may have occurred. Although all patients were treated within a single institution, calendar-time effects and evolving clinical practice may have influenced both perioperative and oncological outcomes and may represent a potential source of temporal confounding. In future multicenter cohorts, adjustment for surgical year or period-based sensitivity analyses could strengthen causal interpretation and address potential temporal bias.

The strengths of this study include its relatively large cohort of octogenarians, use of a validated and practical frailty instrument, and assessment of perioperative morbidity, observed short-term mortality, adjuvant feasibility, and long-term survival. Unlike many prior reports, our analysis provides an integrated evaluation of both surgical and oncological endpoints.

However, several limitations must be acknowledged. The retrospective, single-center design may limit generalizability. Frailty was assessed retrospectively and based on available documentation, which may underestimate frailty prevalence. The mFI-5 predominantly reflects comorbidity burden and does not fully capture cognitive, psychosocial, or functional frailty domains [7]. Data on sarcopenia, formal nutritional indices, and comprehensive geriatric assessment were limited. In addition, the number of variables included in multivariable models may be high relative to the number of outcome events, raising the possibility of model overfitting; therefore, adjusted estimates should be interpreted with caution. Finally, heterogeneity in postoperative care pathways may have influenced outcomes.

Despite these limitations, the consistent associations observed across multiple clinically relevant endpoints suggest that frailty remains an important risk marker for octogenarians undergoing gastrointestinal cancer surgery. Future studies should prospectively validate these findings in multicenter cohorts, integrate more comprehensive geriatric assessments, and evaluate whether targeted prehabilitation and optimization strategies can mitigate the adverse impact of frailty. Incorporating frailty into ERAS pathways and assessing the cost-effectiveness of routine frailty screening should also be priorities.

In conclusion, this study demonstrates that frailty, as defined by the mFI-5, is a key determinant of perioperative complications, observed short-term mortality, adjuvant therapy feasibility, and long-term survival in octogenarian patients undergoing colorectal or gastric cancer surgery. Routine frailty assessment may facilitate risk stratification, support shared decision-making, and help tailor treatment strategies to improve outcomes in this vulnerable and growing patient population.

## Data Availability

The datasets generated and/or analyzed during the current study are not publicly available due to institutional patient-privacy regulations but are available from the corresponding author on reasonable request.

## Abbreviations

BMI: Body mass index
ICU: Intensive care unit
CDC: Centers for Disease Control and Prevention
AJCC: American Joint Committee on Cancer
ERAS: Enhanced Recovery After Surgery
mFI-5: 5-item Modified Frailty Index
OS: Overall survival
RFS: Recurrence-free survival
